# MR microscopy of the human fetal upper extremity – a proof-of-principle study

**DOI:** 10.1186/s12861-016-0123-z

**Published:** 2016-06-18

**Authors:** Inga Langner, Thomas Stahnke, Oliver Stachs, Tobias Lindner, Jens-Peter Kühn, Simon Kim, Andreas Wree, Soenke Langner

**Affiliations:** Division of Hand Surgery and Functional Microsurgery, Department of Trauma and Reconstructive Surgery, University Medicine Greifswald, Greifswald, Germany; Department of Ophthalmology, Rostock University Medical Center, Rostock, Germany; Core Facility Multimodal Small Animal Imaging, Rostock University Medical Center, Rostock, Germany; Department of Anatomy, Rostock University Medical Center, Rostock, Germany; Department of Diagnostic Radiology and Neuroradiology, University Medicine Greifswald, Ferdinand-Sauerbruch-Str. 1, 17475 Greifswald, Germany

**Keywords:** Upper extremity, Fetal development, Ultra high-field MRI, MR microscopy

## Abstract

**Background:**

Current knowledge of the human fetal and embryonic development relies on early descriptive studies of humans and from experimental studies of laboratory animals and embryos. Taking the upper extremity as an example, this study explores the potential of magnetic resonance microscopy (MRM) for the assessment of the development of the fetal upper extremity and discusses its correlation with histological findings.

**Methods:**

*Ex vivo* MRM at 7.1 T (Clin Scan, Bruker Biospin, Germany) was performed in 10 human specimens at 8 to 12 weeks of gestational age (GA). In-plane resolution was 20 μm with a slice thickness of 70 μm. MRM was followed by histological work-up of the specimens. MRM images were then correlated with conventional histology with a focus on the presence of chondrification and ossification.

**Results:**

Ossification of the upper human extremity is detectable at 8 weeks GA in the humerus and the long bones of the forearm. There is excellent correlation for location and size of ossification between MRM and conventional histology. MRM imaging is in accordance with historical studies.

**Conclusion:**

*Ex vivo* MRM for the non-invasive assessment of the embryonic and fetal development of the upper human extremity is feasible. It may provide an accurate complementary tool for the evaluation of embryological development.

## Background

Embryogensis of the human upper limb starts with the formation of the upper limb bud, which is the lateral migration of two layers of mesoderm and an outgrowth into the overlying ectoderm [1–3]. The limb bud appears at embryonic stage 12 (4.5 weeks or 26 days after fertilization). The first vessels within the limb bud appear at stage 13 (31 days) and the hand plate itself is visible at stage 15 (33 days) with completed separation of the digital rays at stage 22 (54 days) [4].

Most of the current understanding of upper extremity development is derived from early descriptive studies of humans and from experimental studies of laboratory animals and embryos [2, 5–7].

Magnetic resonance imaging (MRI) provides excellent soft tissue contrast with high resolution and has multiplanar imaging capability [8]. Furthermore, it is a well-established technique for prenatal imaging, e.g., the detection of fetal brain abnormalities in utero [9]. Ultra-high field magnetic resonance microscopy (MRM) allows acquisition of MR images with submillimeter spatial resolution [8, 10]. Taking the upper extremity as an example, this study explores the potential of MRM for the assessment of the development of the fetal upper extremity and discusses its correlation with histological findings.

## Methods

MRM was performed *ex vivo* in 10 fetal human specimens of the upper extremity. The specimens were 8 to 12 weeks of gestational age (GA) as determined by prenatal ultrasound and were obtained from medically indicated or spontaneous abortions after getting informed written consent from the patients following tenets of the Declaration of Helsinki. The study was approved by the Institutional Review Board (Ethics committee at the University of Rostock, Reference: A200947).

*Ex vivo* MRM was performed on a 7.1 Tesla (T) micro-MRI scanner (ClinScan, Bruker Biospin, Ettlingen, Germany) with a bore size of 13 cm using a small surface loop coil with 1 cm diameter (s1 coil, Bruker Biospin, Ettlingen, Germany) for signal detection. Before MRM the specimen were fixated in formalin (4 %). For MRM, each specimen was placed in an Eppendorf tube filled with 0.9 % saline solution, and the tube was placed in the coil that the entire specimen was covered by the coil for optimal signal detection.

All specimens were examined using exploratory T2-weighted (T2w) localizers followed by high-resolution 3-dimensional T2w turbo spin echo (TSE) sequences in two orthogonal planes. Imaging parameters were: TR 2000 ms, TE 58 ms and field of view (FoV) 20 × 20 mm. With an interpolated matrix of 1024 × 1024 in-plane resolution was 20 μm. Acquisition time was 8 h and 42 min per scan volume. Each volume comprised 96 slices with a slice thickness of 70 μm. Subsequently, some specimens were stained *in toto* using the standard Alizarin – Red S staining method to visualize ossification areas within the upper extremity and then photographed under a microscope at 10× magnification.

For histological workup, the other specimens were embedded in paraffin; sections were cut on a microtome at a thickness of 5 μm according to the image planes used for MRM. For evaluation of calcified bone segments, sections were stained with hematoxylin and eosin (H&E) and azan stain. To identify chondrified segments, sections were stained with Alcian blue and photographed under a microscope at 10× and 40× magnification.

We then correlated conventional histology with MRM in terms of anatomy. Correlation was performed by one board certified radiologist with 15 years of experience in musculoskeletal MRI. Anatomical evaluation focused on the presence of chondrification and ossification of the different bones and bony ultrastructure.

## Results

The 10 specimens examined were obtained between 8 and 12 weeks of GA (*n* = 1 at 8 weeks, *n* = 3 at 9 weeks, *n* = 3 at 10 weeks, *n* = 2 at 11 weeks, and *n* = 2 at 12 weeks).

### Bones

#### 8 weeks of GA

**Fig. 1:**
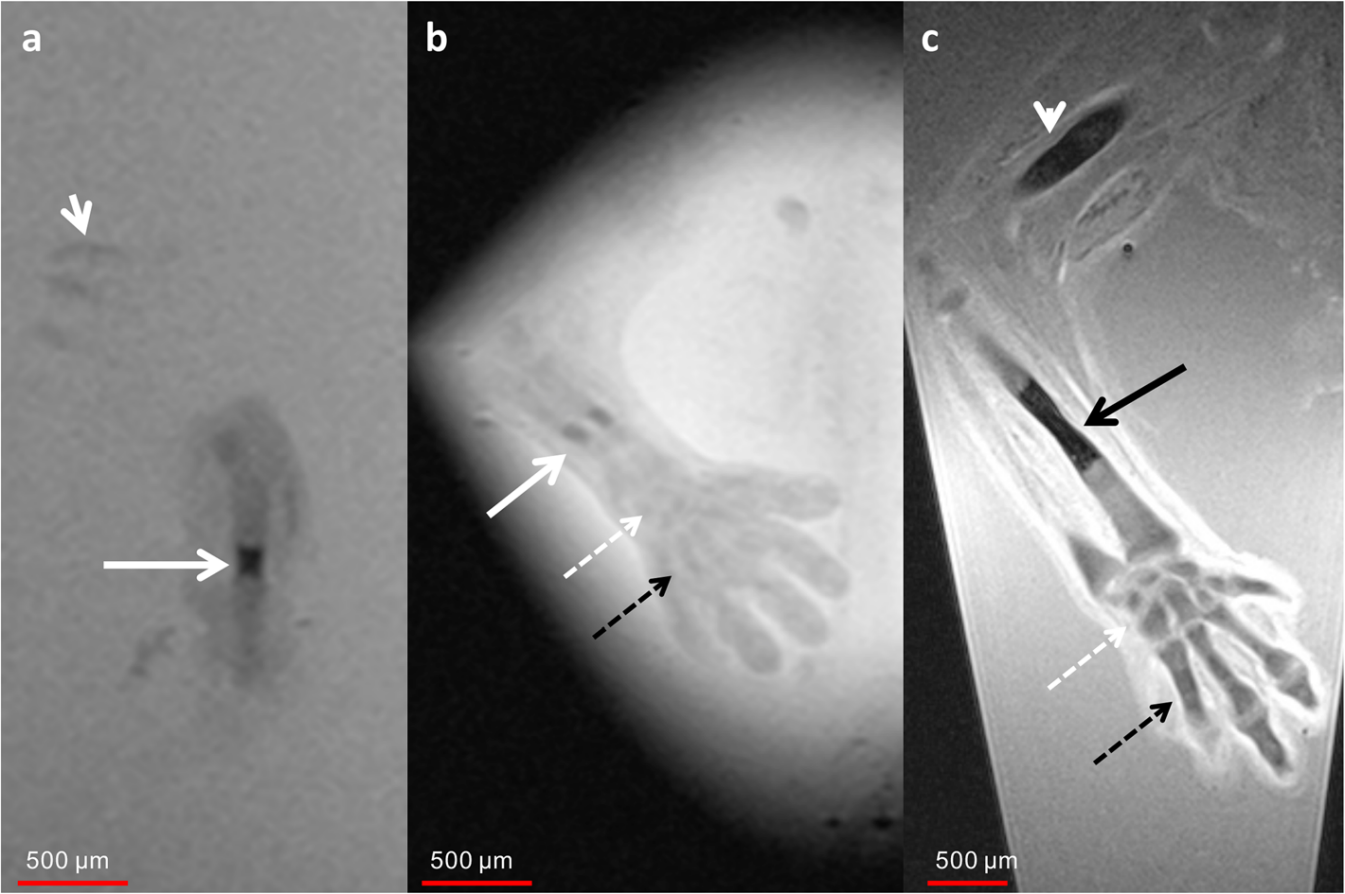
8-week GA specimen. **a** Sagittal T2w image of the humerus of an 8-week GA specimen demonstrates initial ossification within the central part of the diaphysis (*arrow*). Chondrified ribs (*short arrow*). **b** Coronal T2w image of the forearm of the same specimen as in a) demonstrates small ossification centers in the central parts of the radius and ulna (*arrow*). The carpal (*dotted white arrow*) and metacarpal bones (*dotted black arrow*) are already visible as precartilage states. **c** Coronal T2w image of a 9-week GA specimen shows increased size of the ossification centers in humerus (*white arrowhead*) and radius (*black arrow*). The carpal and metacarpal bones demonstrate progressive chondrification and appear hypointense compared to the 8-week GA specimen

At 8 weeks of GA, the chondrified humerus was visible in MRM images with a small ossification center in the middle of the metaphysis (Fig. 1a). Initial ossification with a length of 400 μm was also detectable in the central parts of the radial and ulnar diaphysis (Fig. 1b/c). Mean length of the humerus, radius, and ulna was 6.88 mm, 5.01 mm, and 5.57 mm, respectively. At the level of the carpus, the immature precartilage states of all carpal bones were visible.

#### 9 weeks of GA

**Fig. 2:**
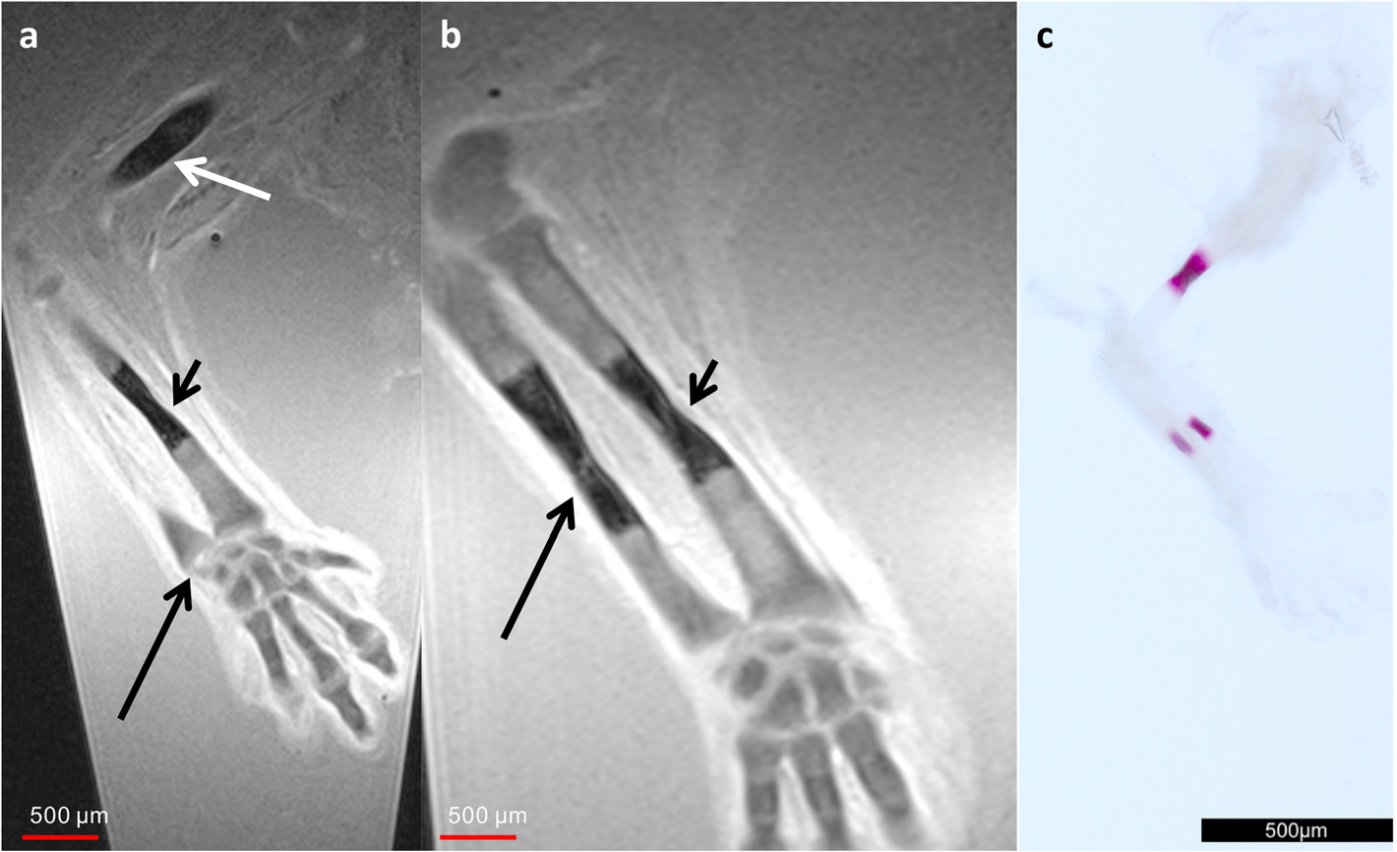
9-week GA specimen. Adjacent coronal T2w images of 9-week GA specimen (**a**, **b**) and photography of the corresponding specimen after *in toto* Alizarin stain (**c**). The ossification centers of the humerus (*white arrow* in **a**), ulna (*black arrow* in **b**) and radius (*short black arrow* in **a** and **b**) appear hypointens on MR images and purple in Alizarin *in toto* stain. The styloid process of the ulna (*long black arrow* in **a**) can be identified but has not retreated from the proximal carpal row

**Fig. 3:**
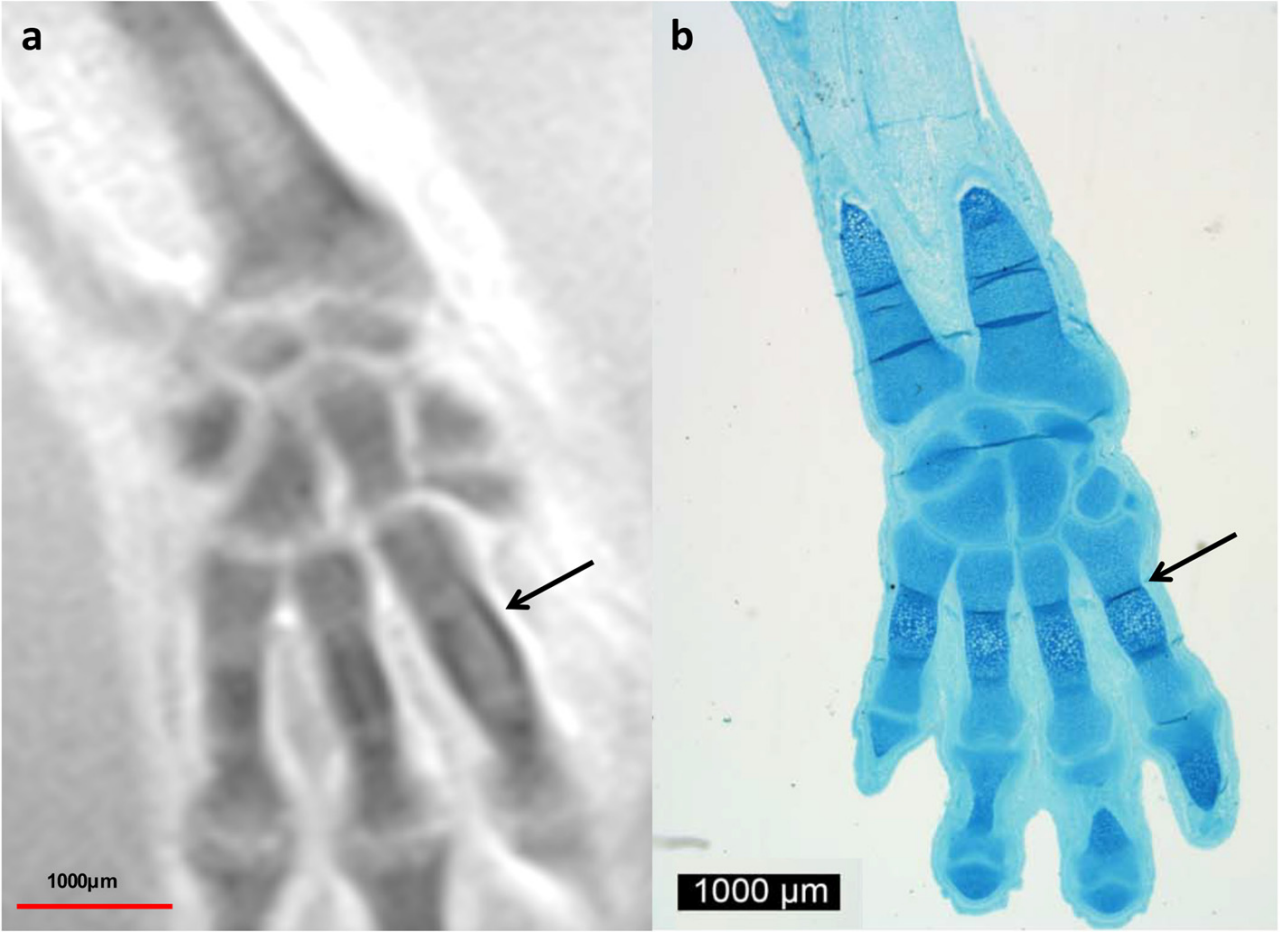
9-week GA specimen. Coronal T2w images of a 9-week GA specimen (**a**) and corresponding Alcian blue stain (**b**). Chondrified centers of all carpal and also initial ossification of the metacarpal bones are clearly visible. Ossification appears pronounced on the ulnar and radial aspect on MRM (**a**, *black arrow*) and in the epiphysial plates in conventional histology (**b**, *black arrow*)

At 9 weeks of GA, all three bones of the upper extremity were clearly visible as isointense chondrified skeletal structures. There was, however, central ossification within the humerus as wells as centrally in the radius and ulna (Fig. 2). Moreover, there was an excellent correlation between MRM images and conventional histology for both size and configuration of the ossification centers, as demonstrated in the *in toto* photographs (Fig. 2). MRM also allowed differentiation between the future cortical and medullary bone formations within the ossification centers (Fig 2b). Chondrification centers for all carpal elements were observed (Fig. 3) and there were signs of initial ossification within the metacarpal bones. Whereas the size and location of these areas correlated well with histology, ossification appeared to be more pronounced on the radial and ulnar aspects of the bones on MRM. In Alcian Blue stain, especially the epiphysial plates appeared prominent. At the level of the wrist, the styloid process of the ulna was differentiated but had not retreated from the proximal carpal row.

#### 11 weeks of GA

**Fig. 4:**
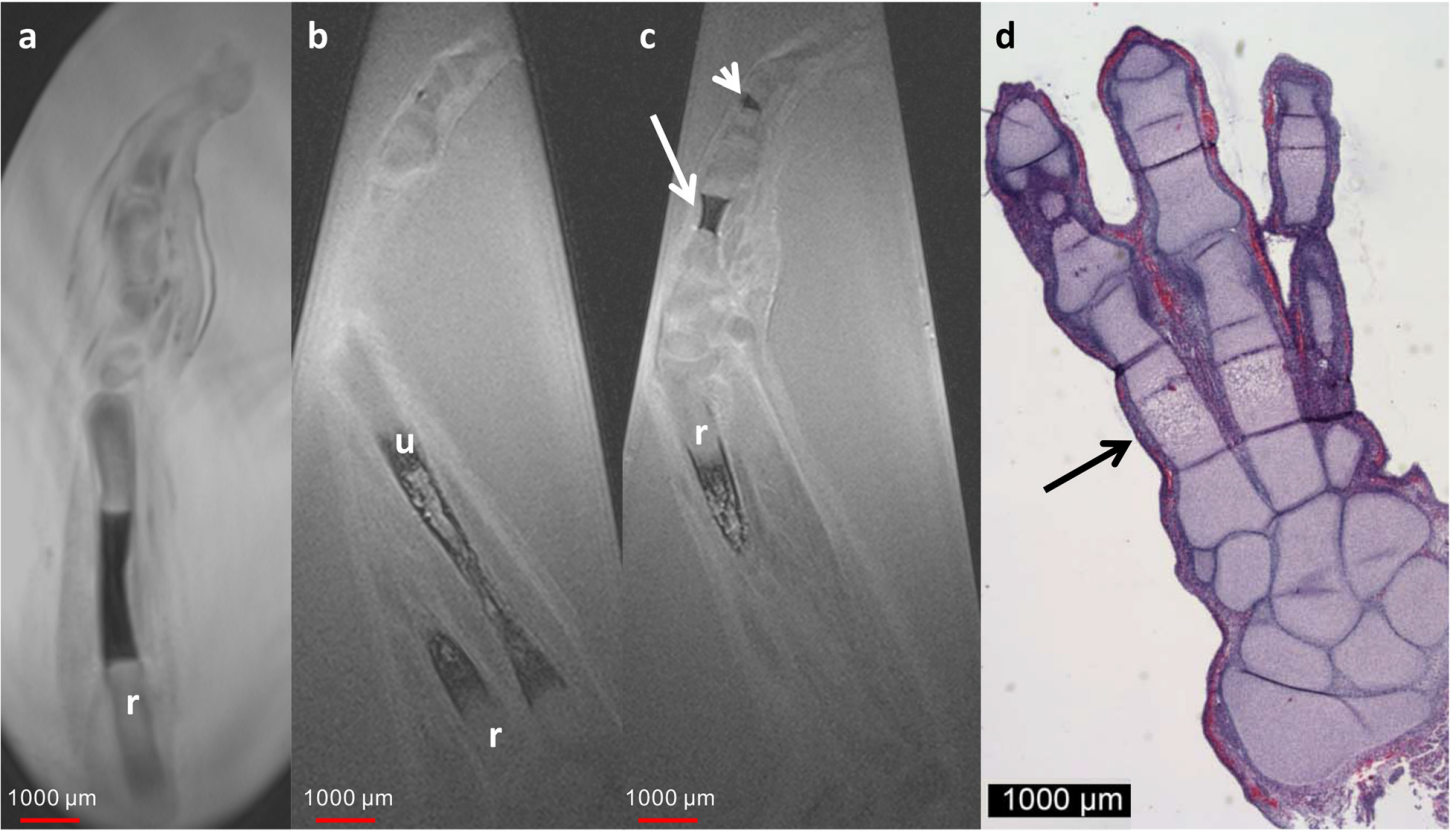
11-week GA specimen. Sagittal T2w image of a 9-week GA specimen (**a**), and sagittal (**b**) and coronal (**c**) T2w image of a 11 week GA specimen demonstrate growth of the ossification centers of radius (r) and ulna (o) with improved differentiation between future cancellous and cortical bone. At 11 week GA ossification of the metacarpal (*arrow* in **c**) and phalangeal bones (*short arrow* in **c**) is visible. **d** Corresponding HE stain with excellent correlation of the size and the location of the ossification centers (*black arrow*)

At 11 weeks of GA MRM demonstrated enlargement of the ossification centers of the radius and ulna (Fig. 4) and allowed improved differentiation of the future medullary cavity and cortical bone. Compared to the prior stages, ossification of the metacarpal and phalangeal bones became apparent. There was good correlation for size and location of the ossification centers with conventional histology (Fig. 4d).

#### 12 weeks of GA

**Fig. 5:**
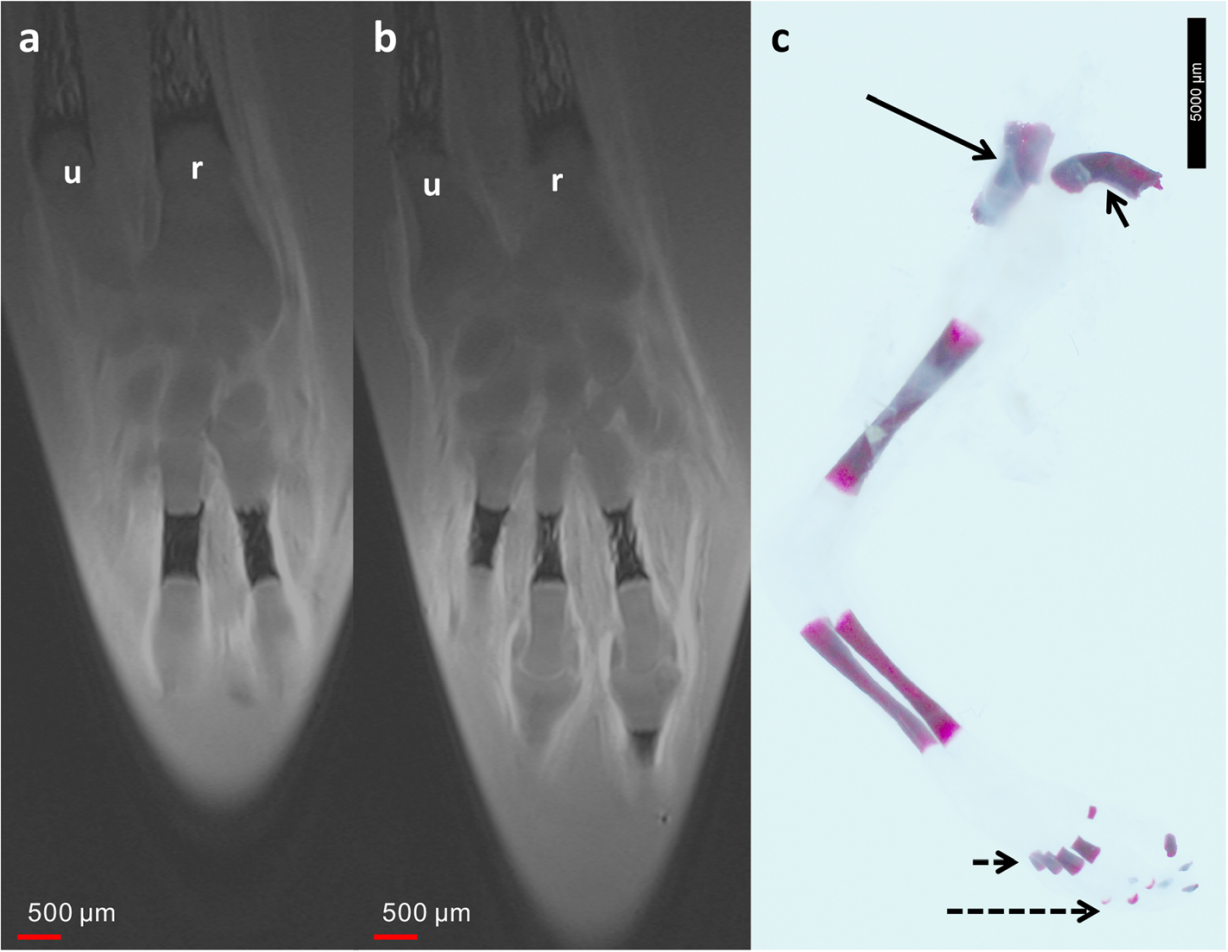
12-week GA specimen. Adjacent coronal T2w images of 12-week GA specimen (**a**, **b**) and photography of *in toto* Alizarin stain (**c**). Compared to the earlier stages there is growth of the ossification centers with excellent correlation between MRM and *in toto* Alizarin stain. Short dotted arrow = metacarpal bones; long dotted arrow = phalanges; r = radius; u = ulna; black arrow in c indicates body of the scapula and short arrow in c indicates body of the clavicle

At this stage, MRM demonstrated ossification of the ulna and radius in the distal parts of the diaphysis and progressive ossification of the metacarpal and phalangeal bones (Fig. 5). In the ulna and radius, MRM again allowed differentiation of the future medullary cavity and cortical bone.

### Muscles, ligaments and tendons

**Fig. 6:**
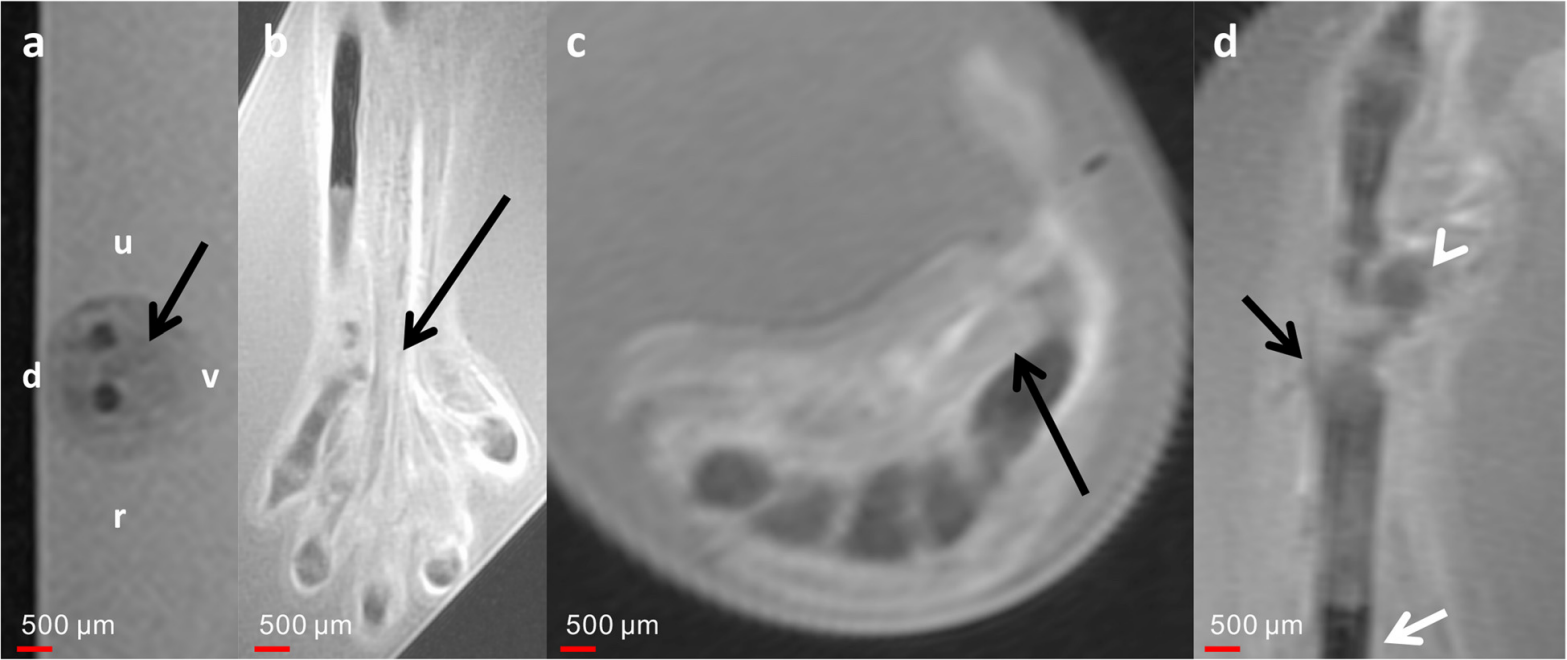
Development of the muscles and tendons at different developmental ages. **a** Axial T2w image of the left forearm of 8-week GA specimen at the mid-diaphyseal level demonstrates the characteristic organization of the muscles along the dorsoventral axis. The ossification centers of radius and ulna appear hypointens compared to the muscles. d = dorsal; v = ventral; u = ulnar; r = radial; white arrow = flexor digitorum muscles. **b** Coronal T2w image of a 9-week GA specimen demonstrates the course of the flexor digitorum tendons. **c** Axial T2w image of a 10-week GA specimen at the level of the thenar demonstrates the course of the short palmar muscles of the thumb. **d** Sagittal T2w image of early 9-week GA specimen with the course of the extensor carpi radialis longus tendon (*black arrow*) to the base of the second metacarpal bone. White arrow indicates ossification center of the radius; white arrowhead indicates scaphoid

**Fig. 7:**
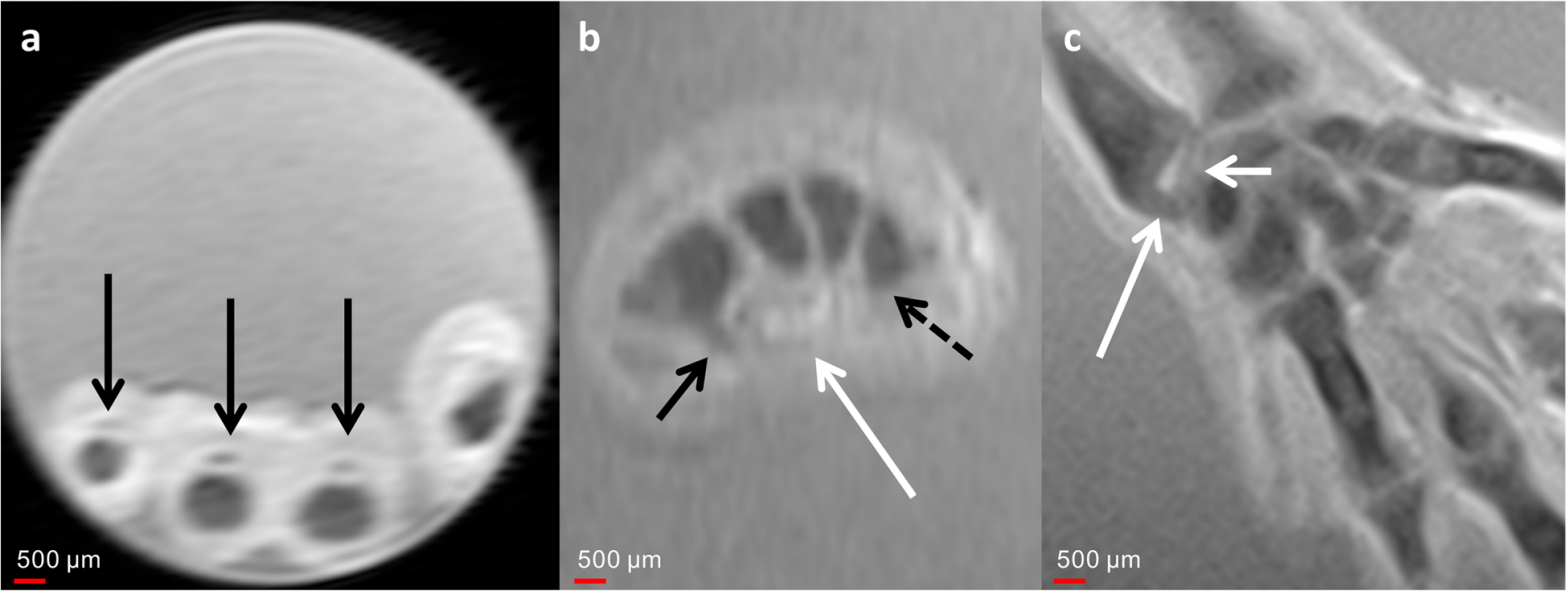
Development of the muscles and ligamentous structures of the wrist at different ages. **a** Axial T2w image of 9-week GA specimen at the level of the base of the proximal phalanges demonstrates the course of the flexor tendons (*black arrow*). **b** Secondarily reconstructed axial T2w image of a 10-week GA specimen demonstrates the transverse carpal ligament (*white arrow*) of the carpal canal at the hamate (*black arrow*) and the trapezoid (*dotted arrow*). **c** Coronal Tw2 image of a 9-week GA specimen. The styloid process of the ulna has not retreated from the triquetrum (*long white arrow*). The articular disc (*short white arrow*) already demonstrates its definite morphology

At 8 weeks of GA, muscles could already be differentiated at the level of the forearm (Fig. 6a). At 9 weeks of GA, the flexor muscles of the forearm could be differentiated and the tendons of the flexor digitorum profundus and superficial muscles could be traced all the way to their insertion (Fig. 7). The intrinsic muscles of the hand were first be differentiated in the late 8th and early 9th week of GA. MRM also allowed the visualization of the ligamentous parts of the wrist. The boundaries of the carpal tunnel became visible at 10 weeks of GA, whereas the future morphology of the triangular fibrocartilaginous complex of the ulna was already delineated at 9 weeks of GA.

## Discussion

The embryogenic period has been divided into 23 developmental stages by O’Rahilly using external and internal morphologic criteria [6, 11] and is followed by the fetal period. The development and morphogenesis of the human upper extremity have been widely studied [2, 5, 6]. However, current knowledge relies on early descriptive studies in human, experimental studies in laboratory animals, and histological work-up of embryos [2, 5–7]. Ultra-high field MRI with improved spatial resolution of less than 100 μm is known as MR microscopy (MRM) [8, 10, 12]. The present study shows that MRM of the upper human extremity is feasible in the late embryonic and early fetal period and correlates well with conventional histology. We achieved an in-plane resolution of 20 × 20 μm and a slice thickness of 70 μm, which is better than in previous reports [8, 12].

Our MRM images demonstrate ossification in the midpart of the humerus at a GA of 8 weeks (O’Rahilly stage 22/23). This is in concordance with previous reports. Czerwinski et al. observed bone formation of the humerus in fetuses with a mean crown-rump length (C-R length) of 20.34 mm, corresponding to a GA of 7 to 8 weeks. Furthermore, there was a good correlation for both, length of the humerus and length of the ossification centers within the humerus, between our results and the findings of Czerwinski et al. [5]. MRM of different specimens at different time points of development demonstrates the growth of the bones and the ossifications, also in good correlation to previous studies [5].

Several studies have investigated chondrification of the forearm, the metacarpal and carpal bones [6, 11, 13]. The latter is classically considered to be a process starting with the capitate and ending with the pisiform [2, 6]. Ossification of the carpal bones however occurs after birth. Our observations agree with recent reports [6]. Premature chondrified structures of the carpal bones can be identified by MRM at 8 weeks of GA. At the beginning of the fetal period, the carpal bones show decreased T2 signal intensity, indicating maturation of the cartilage [14] and that ossification of the metacarpal bones and phalanges has started.

Like in vivo MRI, *ex vivo* MRM allows visualization of the soft tissues. As described by Hita-Contreras et al. [6], differentiation of the muscles of the forearm and the wrist is possible at 8 weeks of GA. However, contrary to their findings, we could identify the extensor carpi radialis muscle at stage 23 and the beginning of the 9th week of GA (Fig. 6d).

Our study has several limitations. The first limitation is the missing crown-rump length. This is due to the origin of the specimens and that only fragmented specimens were available. Therefore, we had to rely on the gestation age determined by prenatal ultrasound for the estimation of GA. However, this is a limitation our study shares with other studies [12] unless the study is based on embryo collections [6]. Nonetheless, our MRM findings at different developmental ages correlate well with published results [5, 6]. Furthermore, the sample size was too small to analyze for sex-related differences [5]. Another limitation is the long acquisition time of MRM. While this is a major issue for in vivo imaging due to motion artifacts, it only plays a minor role in *ex vivo* imaging [8, 15]. Our protocol allows MRM to be performed prior to histopathology without altering routine work-up of the specimen. Due to the fixation in formalin, muscles and the interposed fatty tissue demonstrated only very small differences in signal intensity (Figs. 6 and 7), which might have degraded differentiation of tissue structures. This limitation can be overcome by optimizing specimen preparation [15]. While the spatial resolution of MRM is still inferior to that of conventional histology [10, 16], its major advantage over histological work-up is its noninvasiveness. In the present study, we only acquired anatomical images. However, MRM is also capable of acquiring functional information [17], which may provide further insights into the ultrastructure of the tissue comparable to *in situ* hybridization techniques or immune stains. Correlating the imaging plane of MRM and conventional histology may be difficult but may be improved by the use of a dedicated positioning device [18]. In the present study, we used a 3D dataset, which can be used to generate secondary multiplanar reconstructions to align MRM and conventional histology.

Although MRM is a noninvasive imaging technique, it can only be performed *ex vivo* due to the small diameter of the bore. With increasing availability of ultra-high field MR systems and improvements in coil technology, MRM may become available for clinical routine imaging. While *ex vivo* MRM allows the construction of a MR-based atlas of normal embryonal and fetal human development, safety concerns regarding MR imaging in these early stages of pregnancy have to be overcome by future studies to apply these imaging techniques in vivo and to use these MR-based atlas for the detection of fetal developmental abnormalities.

## Conclusion

In conclusion, our study shows that *ex vivo* MRM for the assessment of the embryonic development of the upper human extremity is feasible and correlates well with conventional histology. The good correlation between MRM and conventional histology as demonstrated in this study is highly supportive for potential clinical applications of this new imaging technique. With increasing availability of ultra-high field MR-systems, this technique may provide an accurate complementary tool for the evaluation of embryonic development.
